# Extended In Vitro Maturation Enhances Oocyte Developmental Competence but Alters Gene Expression in Bovine Embryos Derived From Oocytes With Slow‐Predicted Nuclear Maturation Speed

**DOI:** 10.1002/mrd.70067

**Published:** 2025-11-18

**Authors:** Thomas Chia‐Tang Ho, Takashi Tanida, Takashi Fujii, Keisuke Koyama

**Affiliations:** ^1^ Laboratory of Theriogenology, Graduate School of Veterinary Science Osaka Metropolitan University Izumisano Japan; ^2^ Laboratory of Veterinary Anatomy, Graduate School of Veterinary Science Osaka Metropolitan University Izumisano Japan; ^3^ Faculty of Agriculture Iwate University Iwate Japan

**Keywords:** fertilization timing, individual culture, machine learning, oocyte maturation, time‐lapse monitoring

## Abstract

To identify the optimal in vitro maturation (IVM) duration for bovine oocytes with different nuclear maturation speeds (NMS), this study assessed how varying IVM durations (24, 28, and 32 h) affect developmental competence and embryo quality in oocytes with fast‐ or slow‐predicted NMS classified via machine learning. Developmental competence was evaluated through cleavage rates, first cleavage timing and patterns, and blastocyst formation under individual culture. Embryo quality was assessed via differential staining of inner cell mass and trophectoderm and expression analysis of quality‐related genes in formed blastocysts. For oocytes with slow‐predicted NMS, extending IVM to 28 h increased cleavage rates and accelerated first cleavage timing (*p* < 0.01). The lower blastocyst formation rates of oocytes with slow‐predicted NMS matured for 24 h improved when IVM reached 28 h, becoming comparable to fast‐predicted NMS oocytes. However, extended IVM decreased expression of pluripotency‐related genes (e.g., *NANOG* and *OCT4*; *p* < 0.01) regardless of predicted NMS. In conclusion, extending IVM duration to 28 h improved developmental competence of slow‐predicted NMS oocytes, highlighting the importance of fertilization timing relative to nuclear maturation completion, though it reduced expression of key pluripotency genes. Individualized IVM protocols based on predicted NMS can enhance bovine embryo production efficiency.

## Graphical abstract

Noninvasive machine learning‐based prediction of nuclear maturation speed (NMS) enables precision optimization of in vitro maturation duration for efficient bovine embryo production. Extending maturation from 24 to 28 h improves cleavage and developmental competence of slow‐predicted NMS oocytes but alters key pluripotency‐related gene expression patterns in formed embryos.

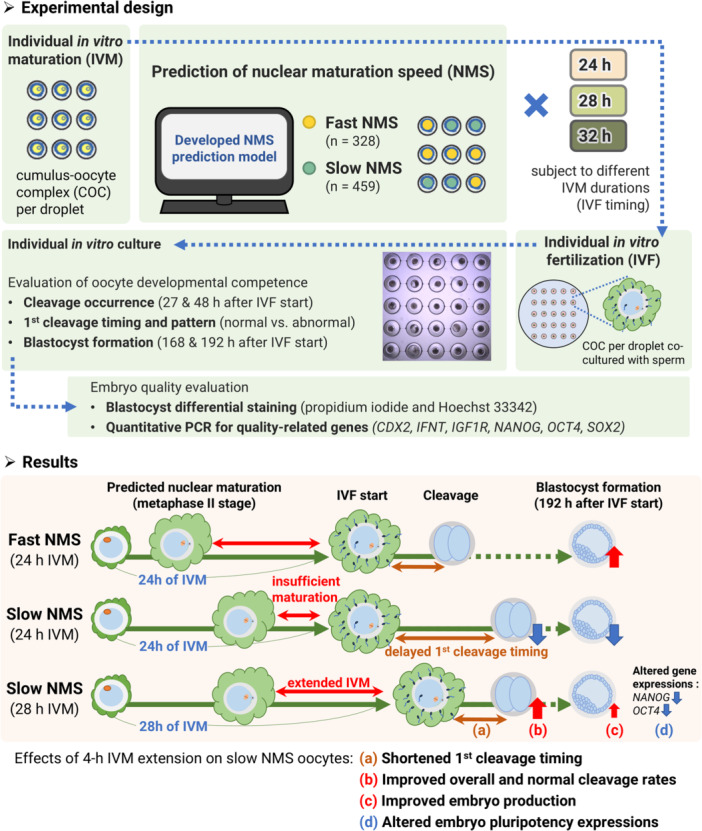

## 1 Introduction

The lack of synchronization between nuclear and cytoplasmic maturation during in vitro maturation (IVM) is considered one of the primary reasons for reduced developmental competence in mammalian oocytes compared to those matured in vivo (Correia et al. 2019; Fulka 1998; Torkashvand et al. 2024). In IVM systems, oocytes undergo spontaneous meiotic resumption soon after collection from follicles (Gilchrist and Thompson 2007), which can lead to insufficient cytoplasmic maturation. Because cytoplasmic maturation involves crucial cellular changes necessary for fertilization and subsequent development (Ferreira et al. 2009; He et al. 2021), ensuring adequate time for this process has become a priority that might be achieved by optimizing IVM duration.

Previous research has suggested that the optimal fertilization timing for bovine in vitro matured oocytes is approximately 12 h after nuclear maturation completion (Koyama et al. 2014). The importance of this interval was also demonstrated by Dominko and First (1997), who found that insufficient time between nuclear maturation (marked by polar body extrusion) and fertilization led to increased chromosomal abnormalities and reduced developmental competence. These findings emphasize the need for proper cytoplasmic maturation, which requires adequate time following nuclear maturation completion before fertilization.

Nuclear maturation speed (NMS) varies significantly among individual bovine oocytes (Koyama et al. 2014; Park et al. 2005; Saeki et al. 1998), posing a fundamental challenge for conventional group culture systems that subject all oocytes to simultaneous fertilization regardless of their individual nuclear maturation progression. Although individual culture offers a potential solution for customized IVF timing based on NMS, assessing nuclear maturation would require the removal of surrounding cumulus cells, which disrupts oocyte‐cumulus cell communication and adversely affects maturation, fertilization, and embryonic development (Dominko and First 1997; Fatehi et al. 2002; Zhang et al. 1995). To overcome this limitation, we established a noninvasive method that predicts NMS through machine learning by analyzing morphological features of cumulus‐oocyte complex (COC) during individual culture (Ho et al. 2023). Using this approach, oocytes were classified into fast‐ or slow‐predicted NMS groups, with prediction accuracy validated by metaphase II (MII) rates at 18 h after IVM start. However, the combined effects of NMS and extended IVM duration on embryo development remain to be elucidated, and clarifying this relationship is essential for defining optimal IVM conditions that accommodate oocytes with different NMS.

While optimizing IVM conditions is crucial for oocyte development, successful in vitro production (IVP) systems must address both embryo productivity and quality. With the aid of time‐lapse monitoring techniques, cleavage dynamics have received considerable attention as noninvasive markers of embryo development and implantation competence both in humans (Çiray et al. 2006; Lundin et al. 2001) and bovine species (Suzuki et al. 2023; Yaacobi‐Artzi et al. 2024). In particular, bovine embryos with slower cleavage timing later than 27 h post‐fertilization exhibit significantly lower viability after transfer (Sugimura et al. 2017), whereas abnormal cleavage patterns are also linked to poor developmental outcomes (Magata et al. 2019; Somfai et al. 2010). The relationship between NMS and these cleavage dynamics remains unexplored and could provide insights of optimized IVF timing.

For embryo quality assessments, the total cell number of the blastocyst, including both inner cell mass (ICM) and trophectoderm (TE) cells, represents a key standard routinely used in embryo evaluation (Van Soom et al. 2001). Higher ICM/TE ratios were observed in in vivo fertilized embryos compared to those produced in vitro (Du et al. 1996; Iwasaki et al. 1990), potentially indicating superior quality. Moreover, gene expression analysis can provide insights into embryo quality and implantation potential (Zolini et al. 2020). Commonly analyzed genes include those involved in TE differentiation, such as *caudal‐type homeobox 2* (*CDX2*) and *interferon tau* (*IFNT*) (Amini et al. 2022; Goissis and Cibelli 2014a); embryo development markers like *insulin‐like growth factor 1 receptor* (*IGF1R*) (Suwik et al. 2021); ICM pluripotency markers, including *Nanog homeobox* (*NANOG*), *POU class 5 homeobox 1* (*OCT4*), and *SRY‐box transcription factor 2* (*SOX2*) (Fujii et al. 2024; Rodríguez‐Alvarez et al. 2010; Velásquez et al. 2019). These genes play crucial roles in embryo development and potentially serve as molecular markers for assessing implantation competence.

Given that oocytes require adequate time after nuclear maturation to complete essential cytoplasmic processes, we proposed two hypotheses. First, extending IVM duration from 24 to 28 h can improve the developmental competence of slow‐predicted NMS oocytes, specifically enhancing their ability to cleave and develop to the blastocyst stage. Second, beyond mere developmental rates, extended IVM can enhance the quality of blastocysts derived from slow‐predicted NMS oocytes, as evaluated through cell allocation patterns and gene expression profiles. To test these hypotheses, we designed three experiments: (1) evaluation of the effects of predicted NMS and extended IVM duration on oocyte developmental competence, including cleavage rates, timing and patterns of first cleavage occurrence, and blastocyst formation; (2) examination of embryo quality from fast‐ and slow‐predicted NMS oocytes under different IVM durations through ICM and TE staining; and (3) analysis of gene expression patterns (*CDX2*, *IFNT*, *IGF1R*, *NANOG*, *OCT4*, *SOX2*) in blastocysts derived from fast‐ and slow‐predicted NMS oocytes cultured under varying IVM durations. This study will demonstrate the importance of optimal fertilization timing and its role in improving oocyte and embryo competence through extended IVM.

## 2 Materials and Methods

### 2.1 Collection of COCs

Ovaries from Japanese Black (JB) beef heifers were obtained from two local abattoirs. The ovaries were transported to the laboratory within 3–6 h after slaughter at approximately 20°C. After washing three times with sterilized saline solution, the ovaries were grouped based on estrous cycle stage (Ireland et al. 1980) into two groups: stages 1 and 4 (S14, paired ovaries without functional corpus luteum [CL]) or stages 2 and 3 (S23, paired ovaries with functional CL). Functional CLs were identified as orange/yellow structures with visible vasculature. S23 ovaries were further divided into S23+ (ovary with CL) and S23− (ovary without CL). Oocyte collection was performed according to this classification, which was subsequently used as a control variable in the statistical analysis of embryo development.

Follicular fluids were aspirated from medium antral follicles (2 to 8 mm in diameter) using commercial oocyte collection medium (OCM; Research Institute for the Functional Peptides, Yamagata, Japan). COCs were selected by a single trained investigator based on morphological appearance: brown, homogeneous cytoplasm surrounded by compact cumulus cells in more than three layers (Nagano et al. 2006), which are considered having normal developmental competence.

### 2.2 IVM and Prediction of NMS

The selected COCs were rinsed with IVM medium (IVMD101; Research Institute for the Functional Peptides) and individually placed into 10 µL droplets of IVMD101 supplemented with FSH (0.02 AU/mL, Antrin R10; Kyoritsu Seiyaku Corporation). The COCs were cultured for different IVM durations of 24, 28, or 32 h under a humidified atmosphere of 5% CO_2_ in air at 38.5°C. Photographs of individual COCs were taken at 0 (beginning of IVM), 12, 15, and 18 h of IVM using an inverted microscope at 100× magnification to extract the needed features for NMS prediction. The COC area was measured by circling the edge of the cumulus investment of each COC using Adobe Photoshop CC 2024 software (Adobe Inc.). Seventeen features such as expansion ratio (the area at each time point divided by the area at 0 h), expansion rate per hour (difference in areas between two time points divided by the number of h passed), and expansion patterns at 18 h after IVM start (categorized as expansion with clusters, expansion without clusters, or barely expanded) were extracted as previously described (Ho et al. 2023). Using the extracted features, oocytes were classified into fast‐ or slow‐predicted NMS groups based on a decision tree–based prediction model developed and validated in our previous study (Ho et al. 2023). This prediction indicates whether an oocyte is expected to reach the MII stage by 18 h after the start of IVM.

### 2.3 IVF and IVC

After maturation, COCs were subjected to 6 h of individual IVF under a humidified atmosphere of 5% O_2_, 5% CO_2_, and 90% N_2_ at 38.5°C. Semen preparation was performed utilizing commercial frozen semen straws from the same JB bull. The straws were thawed at 38.5°C for 40 s and processed using a modified 45%/90% Percoll (Sigma‐Aldrich) gradient method throughout the experiment (Magata et al. 2019; Oliveira et al. 2012). The 90% Percoll medium consisted of 9.98 mg/mL Dulbecco's Modified Eagle Medium (Gibco™ 12100061, Thermo Fisher Scientific Inc., MA, USA), 10 μg/mL gentamicin (G3632, Sigma‐Aldrich), and 6 mM 4‐(2‐hydroxyethyl)‐1‐piperazineethanesulfonic acid (HEPES; H4034, Sigma‐Aldrich). Equal parts of 90% Percoll medium and BO‐SemenPrep™ (IVF Bioscience) were mixed to create the 45% Percoll medium. After gently layering 2 mL of the 45% medium over 2 mL of the 90% Percoll medium, thawed semen was added and centrifuged for 20 min. After removing the supernatant, the remaining sperm were washed by adding 6 mL of IVF medium (IVF100; Research Institute for the Functional Peptides) and centrifuged again for 5 min. For each oocyte, an individual IVF drops (10 µL) was prepared with a final sperm concentration of 2 × 10^6^ sperm/mL. This concentration was achieved by first adjusting the washed sperm pellet to 2 × 10^7^ sperm/mL using a hemocytometer, then adding 1 µL of this adjusted sperm suspension to 9 µL of IVF medium in each drop.

After fertilization, presumptive zygotes were denuded individually using hand‐made glass Pasteur pipettes in OCM‐based denudation medium containing 1 mg/mL of hyaluronidase (H3506, Sigma‐Aldrich) at 37°C. Zygotes were transferred into a microwell culture dish (LinKID micro 25; Dai Nippon Printing Co. Ltd.) containing a drop of 125 µL IVC medium (BO‐IVC™; IVF Bioscience) with 25 microwells. The presumptive zygotes were cultured for 192 h (8 days) from the start of IVF under a humidified atmosphere of 5% O_2_, 5% CO_2_, and 90% N_2_ at 38.5°C. Embryo development was monitored by capturing digital images at specific developmental timepoints: 27 and 48 h after IVF start to assess cleavage occurrence, 168 h for initial blastocyst formation assessment, and 192 h for final evaluation of blastocyst expansion and hatching status.

### 2.4 Timing and Patterns of First Cleavage Occurrence Under Time‐Lapse Monitoring

For a subset of presumptive zygotes, embryo development was monitored using a live cell imaging system (EzScope 101; Blue‐Ray Biotech) with images captured every 20 min to determine the occurrence of the first cleavage and the cleavage patterns. The timing of the first cleavage occurrence was determined by the time of detecting more than one additional blastomere. First cleavage patterns were categorized as either normal or abnormal following established criteria (Somfai et al. 2010). Normal cleavage was defined as the cell cleaved into two equal‐sized blastomeres without signs of fragmentation (Figure 2a). Abnormal cleavage patterns (Figure 2b) were further subcategorized into three distinct types, including direct cleavage into three or more blastomeres without progressing through a two‐cell stage, uneven cleavage resulting in blastomeres of different sizes, and fragmented cleavage with visible cytoplasmic fragments.

### 2.5 Differential Staining of Inner Cell Mass and Trophectoderm Cells in Formed Blastocysts

Day‐8 Blastocysts (192 h after IVF start) were subjected to a modified differential staining method utilizing propidium iodide (PI) and Hoechst 33342 to identify cell allocation of ICM and TE cells, as previously described (Magata et al. 2021; Thouas et al. 2001). Blastocysts were incubated at 38.5°C with RNase (final concentration 10% (v/v), PureLink™ RNase A; Invitrogen) for 60 min by adding 10 µL RNase to the 90 µL IVC medium to reduce RNA interference during PI staining. The blastocysts were then stained with PI (100 μg/mL; Sigma‐Aldrich) in a permeabilizing phosphate‐buffered saline (PBS; 10010023, Thermo Fisher Scientific) solution composed of 0.2% (v/v) Triton X‐100 (Sigma‐Aldrich) and 0.1% (w/v) polyvinylpyrrolidone (PVP; Sigma‐Aldrich), for either 40 s (hatched/hatching blastocysts) or 70 s (blastocysts with zona pellucida). The blastocysts were stained with Hoechst 33342 (1 μg/mL, bisbenzimide H 33342 trihydrochloride; Sigma‐Aldrich) in 4% paraformaldehyde in phosphate buffer solution (FUJIFILM Wako Pure Chemical Corporation, Osaka, Japan) at room temperature for 30 min in the dark. After being washed three times in 0.1%(w/v) PVP in PBS (PVP‐PBS), no more than two blastocysts were individually mounted on each glass slide (S0317; Matsunami Glass Ind. Ltd.) using 8 µL of Fluoromount™ (Diagnostic BioSystems). A cover glass was then placed onto prepared, solidified droplets of a vaseline‐paraffin mixture and gently pressed down until the blastocysts were fixed and could no longer expand. The number of ICM and TE cells was assessed using a confocal laser scanning microscope (FV3000; Olympus). Total cell count (TCC) was determined by counting all cell nuclei (blue, Figure 3a), and TE cell number was determined by counting PI‐positive nuclei (red, Figure 3b). The ICM cell was calculated by subtracting the TE cell count from the TCC, followed by calculation of the ICM/TE ratio.

### 2.6 Quantitative PCR for mRNA Expression in Formed Blastocysts

Blastocysts 192 h after IVF start were washed once in 1% PVP‐PBS and transferred to 0.1% pronase solution (Sigma‐Aldrich) to remove the zona pellucida. When the zona pellucida was barely visible, each blastocyst was washed five times in PVP‐PBS and transferred to PCR tubes containing 5 μL lysis buffer (0.8% Igepal [ICN Biomedical], 5 mM Dithiothreitol [P1171, Invitrogen], and 1 U/μL Ribonuclease Inhibitor [N2111, Promega]). The lysed samples containing blastocyst RNA were snap‐frozen at least 3 times in liquid nitrogen to break down the blastocyst structure and stored at –80°C until reverse transcription.

RNA samples were heated to 80°C for 5 min and then reverse‐transcribed using the QuantiTect Reverse Transcription Kit (Qiagen). The reverse transcription products were validated by standard PCR using the HotStarTaq Master Mix Kit (Qiagen) to confirm the amplification of expected product sizes. Real‐time PCRs were conducted using the QuantStudio 3 system (Applied Biosystems), with products detected using SYBR Green included in the QuantiTect SYBR Green PCR Master Mix (Qiagen). The reverse transcription product was diluted twofold, and 2.5 µL of this diluted product was used for quantification of each targeted gene in duplicates of 10 µL of the PCR solution.

The amplification program was as follows: pre‐incubation at 95°C for 15 min to activate the HotStarTaq DNA Polymerase (Qiagen), followed by 45 cycles of denaturation at 94°C for 15 s, primer annealing at temperatures specified in Table 1 for 30 s, and elongation at 72°C for 30 s. After the last cycle, a melting curve was generated by measuring fluorescence every 0.3°C from 60°C to 95°C to confirm amplification specificity.

**TABLE 1:** List of genes for evaluating formed embryo quality using quantitative reverse transcript PCR.

Gene	GenBank accession	Primers‐forward & reverse	Ta (°C)	Product length (bp)	Reference
*CDX2*	NM_001206299	**F:** *GCCACCATGTACGTGAGCTAC* **R:** *ACATGGTATCCGCCGTAGTC*	61	140	Saadeldin et al. (2012)
*IFNT*	X65539	**F:** *TCCATGAGATGCTCCAGCAGT* **R:** *TGTTGGAGCCCAGTGCAGA*	59	103	Saadeldin et al. (2012)
*IGF1R*	NM_001244612.1	**F:** *GAGTGGAGAAATCTGCGGG* **R:** *AAATGAGCAGGATGTGGAGGT*	57	110	Suwik et al. (2021)
*NANOG*	NM_001025344.1	**F:** *AACAACTGGCCGAGGAATAG* **R:** *AGGAGTGGTTGCTCCAAGAC*	57	193	Khan et al. (2012)
*OCT4*	NM_174580.3	**F:** *GAGAAAGACGTGGTCCGAGTG* **R:** *GACCCAGCAGCCTCAAAATC*	61	101	Suwik et al. (2021)
*SDHA*	NM_174178.2	**F:** *GCAGAACCTGATGCTTTGTG* **R:** *CGTAGGAGAGCGTGTGCTT*	60	185	Goossens et al. (2005)
*SOX2*	NM_001105463.2	**F:** *TGGATCGGCCAGAAGAGGAG* **R:** *CAGGCGAAGAATAATTTGGGGG*	61	89	Suwik et al. (2021)

Abbreviations: bp, base pair; CDX2, caudal‐type homeobox 2; IFNT, interferon tau; IGF1R, insulin‐like growth factor 1 receptor; NANOG, Nanog homeobox; OCT4, POU class 5 homeobox 1; SDHA, succinate dehydrogenase complex flavoprotein subunit A; SOX2, SRY‐box transcription factor 2; Ta, annealing temperature.

The standard curve method was applied to each amplicon using serial dilutions of purified PCR products of known quantity. Standard stocks of each gene were prepared by amplifying each target gene, followed by purification using the QIAquick PCR Purification Kit (Qiagen). The purified products were quantified using a NanoPhotometer (C40, Implen), and diluted to 10 ng/µL for storage at −80°C. Standard curves were generated using serial 10‐fold dilution (ranging from 10^−2^ ng/µL to 10^−13^ ng/µL) and were amplified alongside cDNA samples in every real‐time PCR run. Samples with quantification cycles beyond the lowest standard or without amplifications were considered undetectable and assigned a value of zero. For duplicate samples with a standard deviation larger than one, the analysis was repeated. Samples that continued to show high variation after the second trial were excluded from further analysis. Gene expression was quantified using the Design & Analysis Software (Thermo Fisher Scientific) based on the threshold cycle of detected fluorescence. The expression levels were normalized to the internal reference gene succinate dehydrogenase complex flavoprotein subunit A (*SDHA*), which was selected based on its stable expression in bovine embryos (Goossens et al. 2005).

### 2.7 Experimental Design

First, the effect of extended IVM duration on embryo development between oocytes with different predicted NMS was evaluated. A total of 787 COCs were subjected to either 24 (*n* = 310), 28 (*n* = 201), or 32 h (*n* = 276) of IVM. The morphological features of COC expansion (until 18 h of IVM) were applied to the machine learning decision tree model previously developed for predicting the NMS of each oocyte (Ho et al. 2023), classifying them into fast‐ (*n* = 328) or slow‐predicted NMS groups (*n* = 459). After maturation, the COCs were subjected to IVF and IVC. Although all zygotes were monitored by photo records at 27, 48, 168, and 192 h after IVF start, a majority of COCs (*n* = 634) were monitored through a live cell imaging system to determine the timing and patterns of first cleavage occurrence.

Next, the quality of blastocysts (*n* = 134) derived from COCs in two NMS groups under extended IVM conditions was evaluated by differential staining using PI and Hoechst 33342. Blastocysts showing clear PI staining (*n* = 83) were selected for quality evaluation.

Finally, the effects of extended IVM duration on targeted mRNA expression were examined in formed blastocysts from different NMS groups. For each IVM duration group (24, 28, and 32 h), twelve morphologically normal blastocysts each from fast‐ and slow‐predicted NMS oocytes were selected for RT‐PCR analysis. The relative mRNA abundance was measured for genes related to embryo development (*IGF1R*), TE differentiation (*CDX2* and *IFNT*), and ICM pluripotency (*NANOG*, *OCT4*, and *SOX2*), with expression levels normalized to the housekeeping gene (*SDHA*).

### 2.8 Statistical Analysis

Statistical analyses were performed using R software Version 4.4.0 (R core Team 2021). Statistical significance was defined as *p* < 0.05, while a value of 0.05 ≤ *p* ≤ 0.10 was considered as a tendency.

Before analysis, continuous outcome variables were examined for normality using Shapiro–Wilk test. Log transformation was applied for relative gene expressions (*CDX2*, *IFNT*, *IGF1R*, *NANOG*, *OCT4*, and *SOX2*), TCC, TE cell count, and ICM/TE ratio data when necessary to meet the assumption of normality.

Binomial outcome variables were analyzed using generalized linear models (‘glm’ function with binomial distribution and logit link function). For all inseminated COCs (*n* = 787), we investigated the effects of IVM duration, NMS group, and their interaction (explanatory variables) on cleavage occurrence, blastocyst formation, and hatching/hatched blastocyst formation, with estrous cycle (ovarian origin of each COC; S14, S23+, or S23−) included as a control variable. For cleaved zygotes within 48 h after IVF (*n* = 560), we assessed blastocyst formation at 192 h and hatching/hatched blastocyst formation using the same statistical approach described above.

For zygotes monitored by time‐lapse camera (*n* = 634), normal cleavage occurrence (binary variable) was analyzed using generalized linear models, while first cleavage timing (continuous variable) was analyzed using a linear model (‘glm’ function). All models included IVM duration, NMS group, and their interaction as explanatory variables, and estrous cycle as a control variable.

For cleaved zygotes monitored by time‐lapse camera (*n* = 458), normal cleavage or direct cleavage occurrence as outcome variables were analyzed using generalized linear models with IVM duration, NMS group, and their interaction as explanatory variables, respectively, with estrous cycle included as a control variable.

Linear models (‘lm’ function) were employed to analyze log‐transformed continuous variables including relative gene expressions (*CDX2, IFNT, IGF1R, NANOG, OCT4*, and *SOX2*), TCC, TE cell count, and ICM/TE ratio. The effects of IVM duration, NMS group, and their interaction were assessed, with blastocyst stage (blastocyst, expanded blastocyst, or hatching/hatched blastocyst) included as a covariate.

Post hoc pairwise comparison for binary variables was conducted using the ‘glht’ function (multcomp package), followed by Tukey adjustments, while continuous variables were analyzed using the ‘emmeans’ function with Tukey adjustments as post hoc pairwise comparison.

## 3 Results

### 3.1 Effect of IVM Duration and Predicted NMS on Oocyte Developmental Competence

**FIGURE 1:**
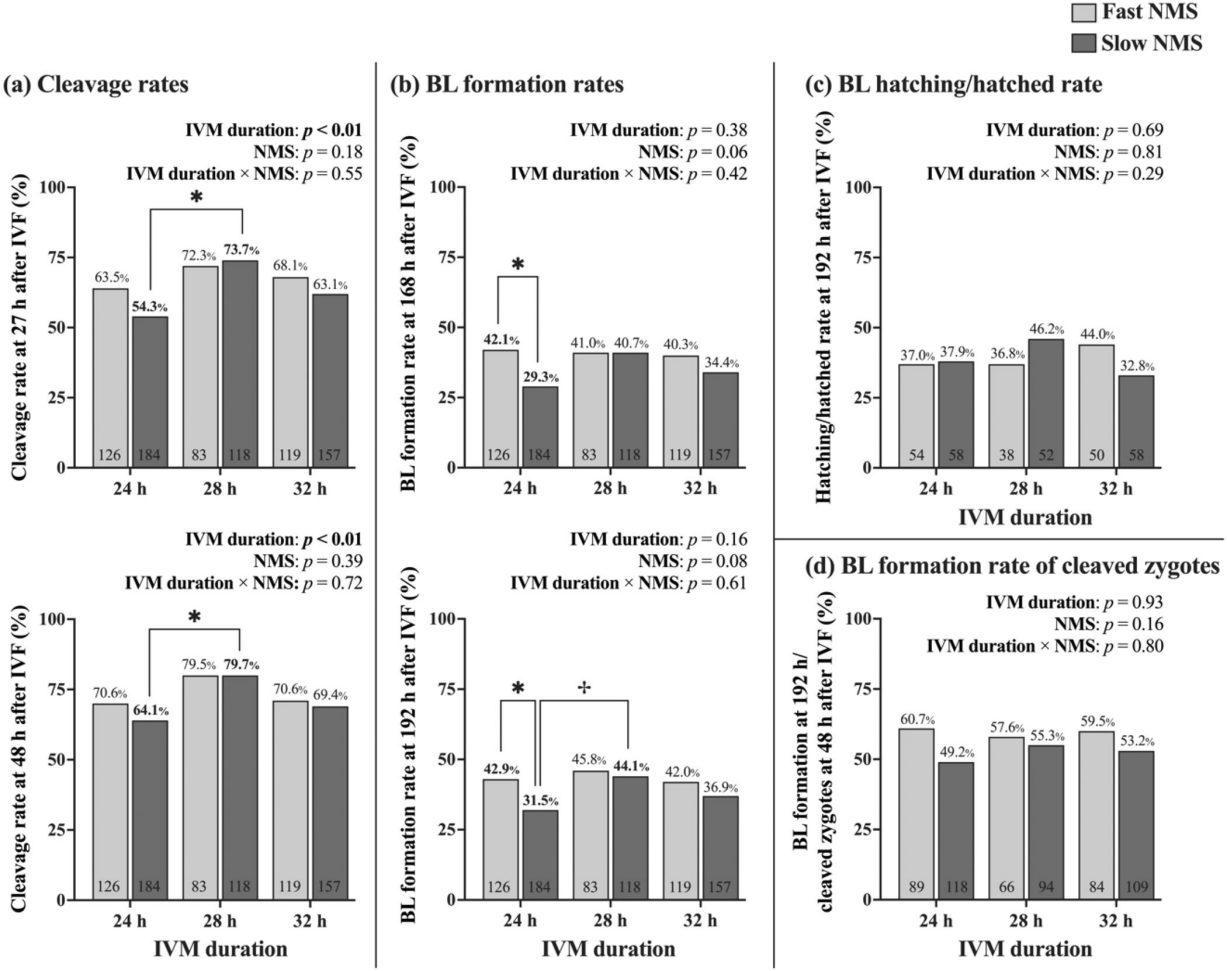
The effect of extended in vitro maturation (IVM) durations on (a) cleavage rates, (b) blastocyst (BL) formation rates, (c) hatching/hatched BL rate, and (d) BL formation rate relative to cleaved zygotes after the in vitro fertilization (IVF) between oocytes with fast‐ and slow‐predicted nuclear maturation speed (NMS). An asterisk (*) indicates a significant difference (*p* < 0.05), and a dagger (†) indicates a tendency toward difference (*p* < 0.10) between comparisons. Numbers within bars indicate the number of oocytes for each category.

In embryos from oocytes with slow‐predicted NMS, cleavage rates at 27 and 48 h after IVF start were higher when IVM was performed for 28 h compared to those matured for 24 h (*p* < 0.01; Figure 1a). In contrast, extending IVM duration did not alter cleavage rates in embryos derived from fast‐predicted NMS oocytes. Within each IVM duration, no significant differences in cleavage rates were observed between NMS groups at either 27 or 48 h post‐IVF.

For blastocyst formation, oocytes with fast‐predicted NMS showed higher rates at 168 h after IVF start compared to those with slow‐predicted NMS when matured for 24 h (*p* < 0.05; Figure 1b). No significant difference between NMS groups was observed when IVM was extended to 28 h. In embryos from slow‐predicted NMS oocytes, extending IVM to 28 h showed a trend toward higher blastocyst formation at 192 h after IVF start compared to 24 h IVM (*p* = 0.077). Neither IVM duration nor NMS group significantly affected three other developmental parameters: the rate of hatching/hatched blastocysts (Figure 1c), the percentage of blastocysts formed at 192 h from cleaved zygotes (assessed at 48 h; Figure 1d), and the percentage of hatching/hatched blastocysts from zygotes cleaved within 48 h after IVF start.

### 3.2 Effect of IVM Duration and Predicted NMS on Cleavage Timing and Patterns Under a Time‐Lapse Monitoring System

**FIGURE 2:**
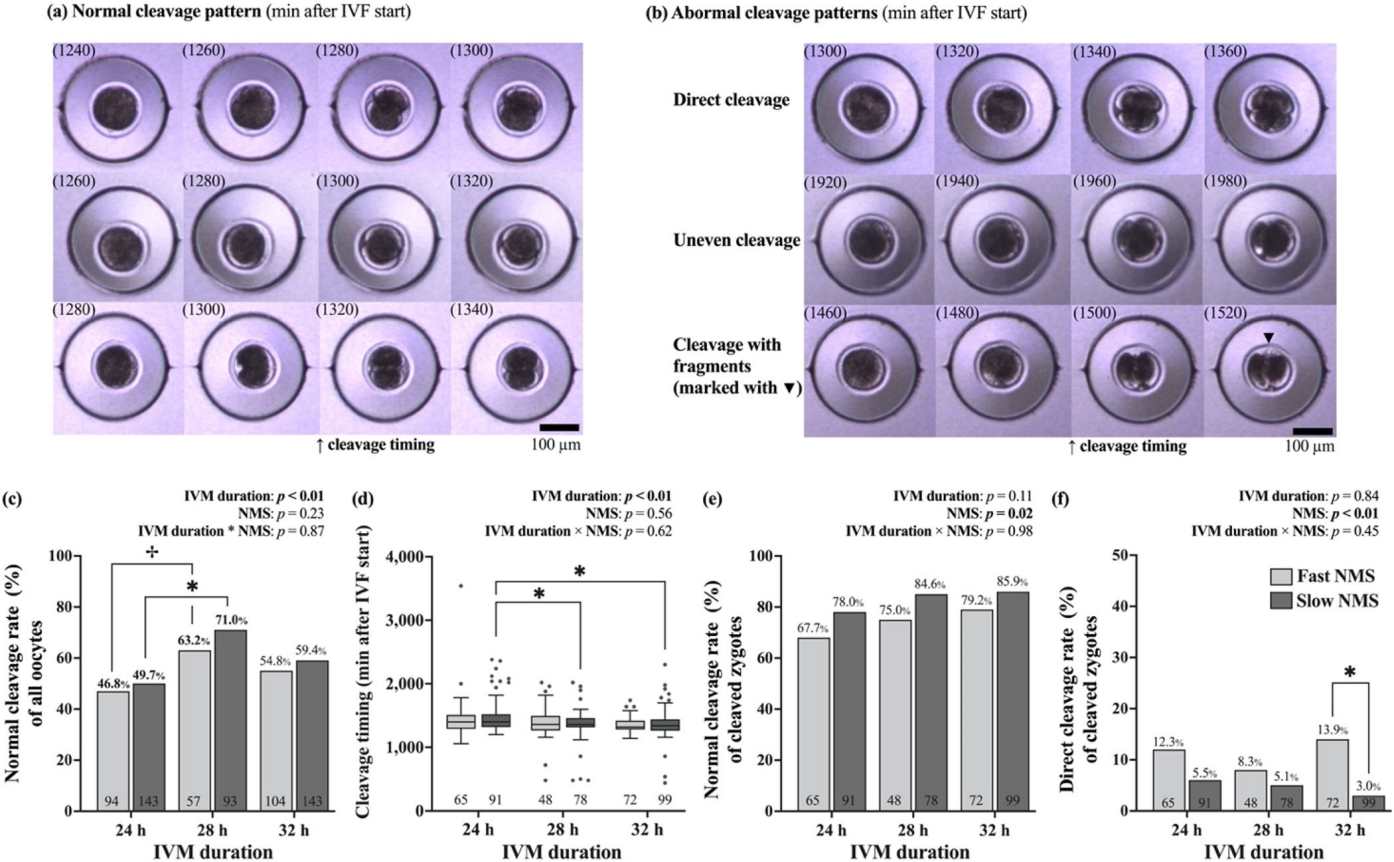
The effect of extended in vitro maturation (IVM) durations on first cleavage pattern and timing between oocytes with fast‐ and slow‐predicted nuclear maturation speed (NMS). Representative images of (a) normal cleavage pattern and (b) abnormal cleavage patterns under a time‐lapse monitoring system every 20 min (numbers inside parentheses indicate minutes after the start of in vitro fertilization [IVF]). (c) Normal cleavage rate of all oocytes between IVM durations and NMS groups. (d) First cleavage timing (min after IVF start), (e) Normal cleavage rate, and (f) direct cleavage rate of cleaved zygotes between IVM durations and NMS groups. An asterisk (*) indicates a significant difference (*p* < 0.05) between comparisons, and a dagger (†) indicates a tendency toward difference (*p* < 0.10) between comparisons. Numbers inside each bar/below each boxplot indicate the number of oocytes/cleaved zygotes analyzed.

The normal cleavage rate was higher in oocytes with slow‐predicted NMS when IVM was performed for 28 h compared to those matured for 24 h (*p* < 0.01; Figure 2c). Similarly, oocytes with fast‐predicted NMS showed a trend toward higher normal cleavage rate when matured for 28 h compared to those matured for 24 h (*p* = 0.058).

Within cleaved zygotes from oocytes with slow‐predicted NMS, first cleavage was delayed in oocytes matured for 24 h compared to those matured for 28 and 32 h (*p* < 0.01; Figure 2d). However, extending IVM to either 28 or 32 h in slow‐predicted NMS oocytes did not significantly affect the proportion of normal cleavage or the incidence of direct cleavage among cleaved zygotes (Figure 2e,f).

Across all IVM durations, the proportion of normal cleavage was higher in cleaved zygotes derived from oocytes with slow‐predicted NMS compared to those from oocytes with fast‐predicted NMS (*p* < 0.05; Figure 2e); however, when analyzed within each IVM duration separately, no significant difference was detected between NMS groups within the same IVM duration. Moreover, the incidence of direct cleavage was higher in cleaved zygotes derived from oocytes with fast‐predicted NMS compared to those from oocytes with slow‐predicted NMS across all IVM durations (*p* < 0.01). When analyzed within each IVM duration separately, this difference was observed only when IVM was performed for 32 h (*p* < 0.01; Figure 2f).

### 3.3 Effect of IVM Duration and Predicted NMS on TCC, TE Cell Number, and ICM/TE Ratio

**FIGURE 3:**
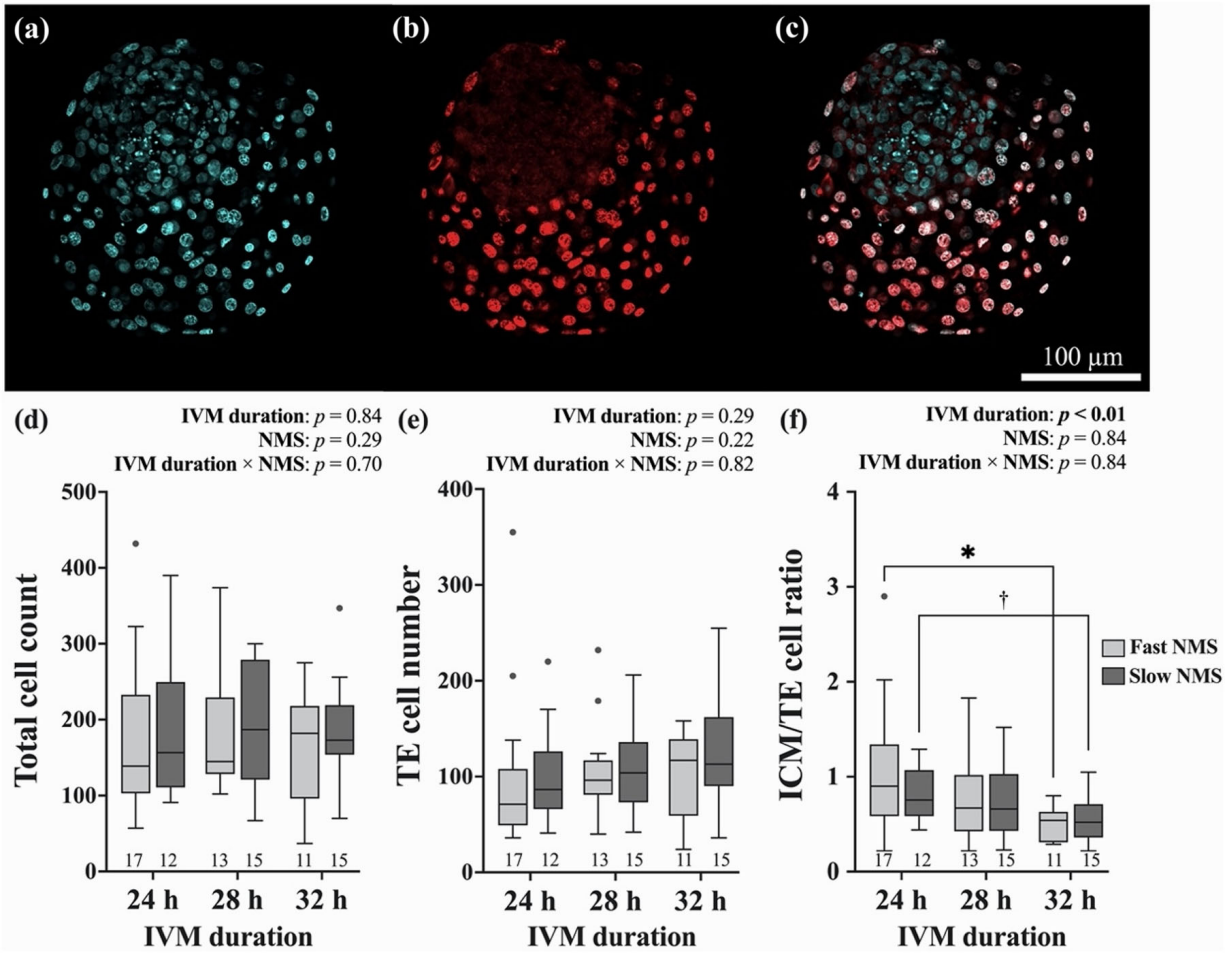
Differential staining of the formed blastocysts from oocytes with different in vitro maturation (IVM) durations and predicted nuclear maturation speed (NMS: fast‐ vs. slow‐predicted). Representative images of blastocyst nuclei stained differentially with Hoechst (a), propidium iodide (b), and their merged overlay (c). Comparison of (d) total cell count, (e) trophectoderm (TE) cell number, and (f) inner cell mass (ICM) to TE cell ratio in blastocysts between IVM durations and NMS groups. An asterisk (*) indicates a significant difference (*p* < 0.05), and a dagger (†) indicates a tendency toward difference (*p* < 0.10) between comparisons. The numbers below each boxplot indicate the number of embryos in each category.

Although no significant differences in TCC or TE cell numbers were observed between NMS groups or across different IVM durations (Figure 3d,e), the effect of IVM duration was found to be significant in ICM/TE ratio (*p* < 0.01; Figure 3f). Extending IVM duration from 24 to 32 h significantly reduced the ICM/TE ratio in embryos derived from fast‐predicted NMS oocytes (*p* < 0.05), with a similar but nonsignificant trend observed in embryos from slow‐predicted NMS oocytes (*p* < 0.10). No significant differences in ICM/TE ratio were observed between NMS groups within any IVM duration.

### 3.4 Effects of IVM Duration and Predicted NMS on Gene Expressions in Blastocysts

**FIGURE 4:**
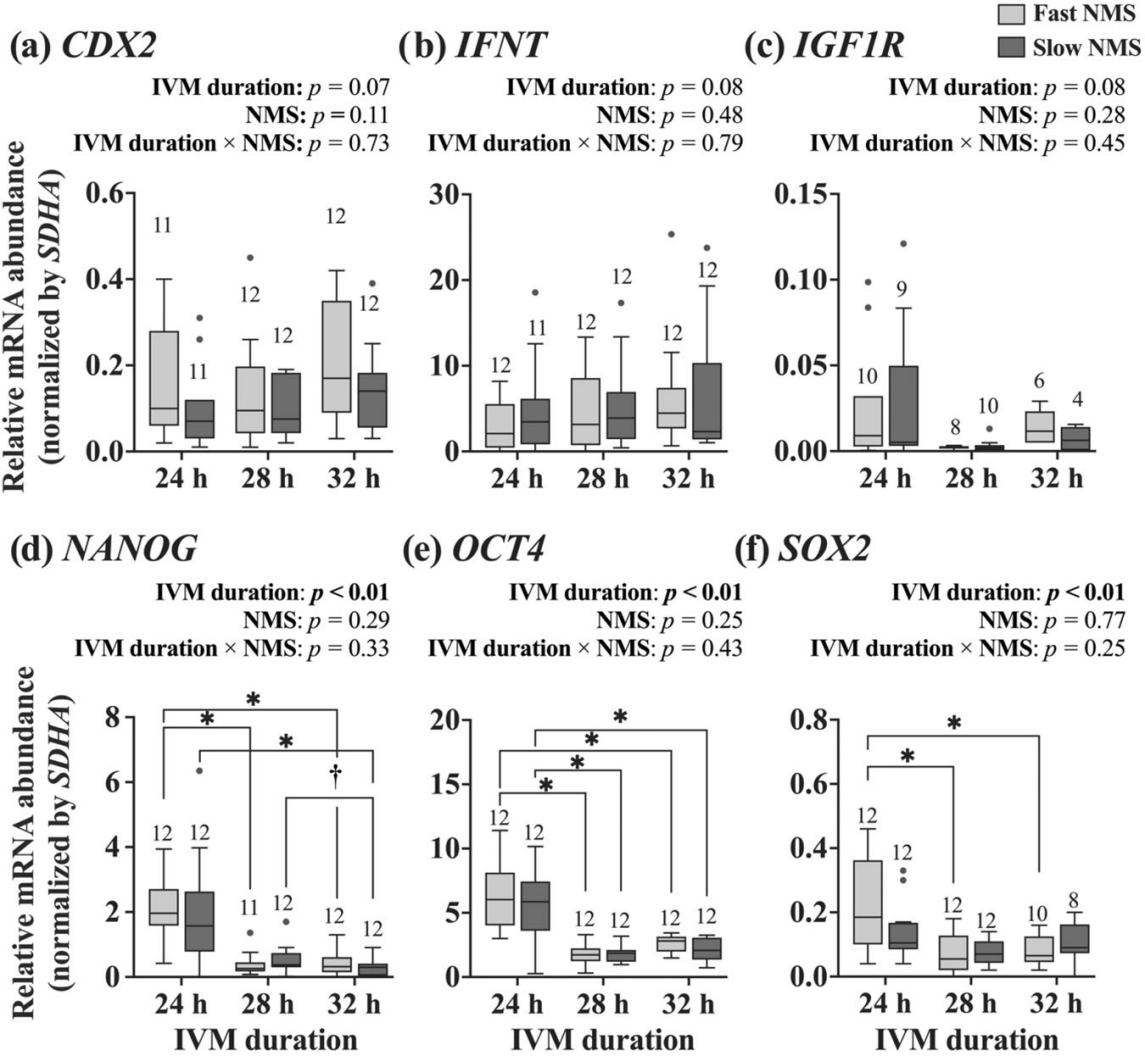
Comparison of the relative mRNA abundance of (a) *caudal‐type homeobox 2* (*CDX2*), (b) *interferon tau* (*IFNT*), (c) *insulin‐like growth factor 1 receptor* (*IGF1R*), (d) *Nanog homeobox* (*NANOG*), (e) *POU class 5 homeobox 1* (*OCT4*), and (f) *SRY‐box transcription factor 2* (*SOX2*) in blastocysts from oocytes with different in vitro maturation (IVM) durations and predicted nuclear maturation speed (NMS; fast‐ vs. slow‐predicted). An asterisk (*) indicates a significant difference (*p* < 0.05), and a dagger (†) indicates a tendency toward difference (*p* < 0.10) between groups. Numbers above boxplots indicate the number of embryos for each category. *SDHA*: *succinate dehydrogenase complex flavoprotein subunit A*.

The main effects of IVM duration showed trends toward significance in the expressions of *CDX2* (Figure 4a), *IFNT* (Figure 4b), and *IGF1R* (Figure 4c; *p* < 0.10); whereas post hoc pairwise comparisons within each NMS group did not reveal statistical differences between different IVM durations. In contrast, the expression levels of *NANOG* (Figure 4d), *OCT4* (Figure 4e), and *SOX2* (Figure 4f) were affected by IVM duration (*p* < 0.01). Among embryos derived from oocytes with fast‐predicted NMS, all three genes exhibited lower mRNA abundance in the 28 and 32 h groups compared to the 24 h group of IVM duration (*p* < 0.01). Within the slow‐predicted NMS group, *NANOG* expression was lower in embryos derived from oocytes matured for 32 h compared to those matured for 24 h (*p* < 0.01). Similarly, *OCT4* expression was reduced in embryos from oocytes matured for both 28 and 32 h compared to those matured for 24 h (*p* < 0.01). No difference was detected in the *SOX2* expression between each IVM durations in embryos from oocytes with slow‐predicted NMS.

## 4 Discussion

Our findings demonstrated improved developmental competence of slow‐predicted NMS oocytes when IVM duration was extended from 24 to 28 h, as evidenced by significantly increased cleavage rates and a tendency toward improved blastocyst yield. These results are consistent with the first hypothesis, indicating that impaired developmental potential in oocytes with slow‐predicted NMS could be mitigated by adjusting suitable IVF timing. When IVM was performed for 24 h, a significant difference in blastocyst formation rate was observed between embryos from fast‐ and slow‐predicted NMS oocytes, supporting the notion that the duration from nuclear maturation to fertilization affects oocyte developmental potential (Koyama et al. 2014). Despite culture variations between studies, our findings offer insight into the inconsistencies in optimal IVM durations reported in group culture systems (Merton et al. 2012; Ward et al. 2002). Extending IVM by 4 h increased blastocyst yield in slow‐predicted NMS oocytes from 31.5% to 44.1%, indicating that extending IVM duration can markedly improve the developmental potential of this population. Accordingly, incorporating NMS prediction into IVP systems, combined with refinements in individual culture conditions and NMS prediction accuracy, holds great potential for achieving higher embryo production efficiency.

In oocytes with slow‐predicted NMS, extending IVM to 28 h improved not only cleavage and blastocyst formation rates but also the proportion of normal cleavage, suggesting that this extended duration helps reduce the risk of abnormal fertilization. Moreover, the timing of the first cleavage was significantly shortened in slow‐predicted NMS oocytes after IVM extension, indicating a faster fertilization process. As a result, extended IVM is likely to enhance fertilization competence in oocytes with slow‐predicted NMS by allowing adequate time for cytoplasmic maturation. Among various cytoplasmic events that occur during maturation, the proper arrangement of cortical granules is particularly crucial for successful fertilization (Ferreira et al. 2009). These specialized secretory vesicles undergo a precise migration process during IVM: they are initially distributed in clusters throughout the cytoplasm at the germinal vesicle stage (Hosoe and Shioya 1997) and subsequently migrated towards specific positions beneath the inner surface of the plasma membrane as oocyte progress to MII stage (Niimura and Hosoe 1995). This migration is essential for successful fertilization; these cortical granules contain enzymes that could modify zona pellucida for preventing polyspermy (Duque 2002; Hosoe and Shioya 1997). Notably, previous research has shown that after 24 h of IVM, 27% of bovine oocytes still exhibit incomplete cortical granule migration (Niimura and Hosoe 1995), which could explain, at least in part, the reduced fertilization competence we observed in slow‐predicted NMS oocytes matured for 24 h. Future studies investigating the relationship between extended IVM and cortical granule distribution patterns would provide mechanistic insights into the improved fertilization competence observed in this study.

While extended IVM in slow‐predicted NMS oocytes clearly improved fertilization competence, its effects on embryo quality showed inconsistent results. As earlier cleavage timing correlates with improved implantation outcomes after embryo transfer in bovine species (Sugimura et al. 2017), the shortened first cleavage timing in slow‐predicted NMS oocytes suggests a higher implantation competence when IVM was extended from 24 to 28 h. This improvement may be explained by the association between delayed first cleavage timing and the higher incidence of chromosomal abnormalities (Sugimura et al. 2012), which is also reported in cases with insufficient intervals between nuclear maturation and fertilization (Dominko and First 1997). Even though these findings reveal potential beneficial effects of extended maturation on embryo quality, extending IVM appears to have a limited effect on improving embryo quality post‐fertilization. In terms of blastocyst cell allocation, we found no significant changes in total cell counts or ICM/TE ratios in blastocysts derived from slow‐predicted NMS oocytes matured for 28 h compared to 24 h. More concerning, the extended IVM resulted in significantly decreased expression of two key developmental genes in formed blastocysts: *NANOG* and *OCT4*, which play crucial roles in maintaining ICM pluripotency (Degrelle et al. 2005). Functional studies have demonstrated the critical roles of these genes in embryo development: knockout of *OCT4* in bovine embryos leads to reduced *NANOG* expression and impaired blastocyst development (Simmet et al. 2018), while *NANOG* knockout in bovine embryos results in decreased total cell numbers in blastocysts (Springer et al. 2021). The importance of these genes is further supported by studies showing the higher *NANOG* expression in vivo‐derived embryos comparing to IVP embryos (Fujii et al. 2024). Nevertheless, these seemingly contradictory results—earlier first cleavage timing but reduced expression of key developmental genes—can be interpreted by examining the temporal dynamics of gene expression patterns during normal embryonic differentiation. As the quantification cycle values of the reference gene (*SDHA*) remained stable with extended IVM culture, RNA degradation can be ruled out as a primary cause of the observed expression changes. This reduction in pluripotency‐related gene expression after extended IVM likely represents accelerated developmental progression rather than compromised embryo quality, based on several lines of evidence. First, these genes naturally peak at the morula stage and decline during later development toward the blastocyst stage (Khan et al. 2012). Second, similar patterns of reduced pluripotency gene expression occur naturally during embryo development, as demonstrated when comparing embryos cultured for 9 versus 13 days (Velásquez et al. 2019) as well as 7 versus 9 days (Simmet et al. 2022). Third, this concordance between gene expression pattern and earlier cleavage timing supports the interpretation of accelerated development rather than quality impairment. This view is further strengthened by the absence of negative effects on morphological parameters such as total cell numbers and ICM/TE ratios in embryos from extended IVM oocytes. Although the extended IVM duration could be accelerating normal developmental processes rather than compromising embryo quality, this hypothesis requires further examination through direct assessment of implantation outcomes.

Unlike *NANOG* and *OCT4*, decreased *SOX2* expression following extended IVM was observed exclusively in embryos derived from oocytes with fast‐predicted NMS. This suggests that fast‐predicted NMS oocytes respond to extended maturation with a comprehensive downregulation of multiple pluripotency genes examined, whereas slow‐predicted NMS oocytes exhibit a selective response pattern, maintaining stable *SOX2* expression despite decreases in other pluripotency markers. As a key component of the pluripotency network primarily localized in ICM after immunostaining (Goissis and Cibelli 2014b), *SOX2* plays crucial roles in both ICM and TE development. Its importance in TE formation has been demonstrated in murine research, where *SOX2* small interfering RNA injection resulted in reduced expression of TE‐associated markers and decreased blastocyst formation rates (Keramari et al. 2010). Additionally, lower *SOX2* expression has been associated with delayed blastocyst formation and reduced embryo development in late‐cleaved porcine zygotes (Sun et al. 2024). Evaluating changes in *SOX2* expression in response to extended IVM may help elucidate the differential molecular mechanisms between oocytes with distinct NMS.

Rescuing the developmental competence of slow‐predicted NMS oocytes remains challenging, as demonstrated by the enhanced embryo development yet accompanied by decreased expression of pluripotency‐associated genes. Nevertheless, this approach to NMS prediction offers a targeted strategy to improve the developmental competence of slow‐predicted NMS oocytes that enables early identification and intervention. While simply extending IVM duration is one way to enhance cytoplasmic maturation, various supplementation strategies could also assist the development of slow‐predicted NMS oocytes without compromising gene expression. For instance, supplementation with fibroblast growth factor 10—one of the oocyte secreted factors (Caixeta et al. 2013)—during IVM has been reported to improve cleavage competence and the expression of pluripotency‐related genes in porcine embryos derived somatic cell nuclear transfer (Son et al. 2018). Furthermore, it remains unclear whether NMS varies under simulated meiotic resumption using cAMP modulators, potentially resulting in suboptimal fertilization and inconsistent results when applying systems of simulated physiological oocyte maturation (Leal et al. 2022). Performing optimized fertilization based on NMS under these culture systems may further improve developmental competence while addressing the potential risks of extended IVM duration. Finally, the mechanisms underlying NMS variation remain unclear, but their clarification is essential for refining culture strategies tailored to inherent oocyte heterogeneity. As ovaries were obtained from abattoirs, the 3–6 h post‐slaughter collection interval—while including the 3–5 h reported as optimal (Blondin et al. 1997)—cannot be ruled out as a factor influencing oocyte developmental competence. Similarly, variability in the size of aspirated follicles (2–8 mm) may also contribute to differences in oocyte quality. These factors highlight the practical challenge of working with a diverse oocyte population, which in turn underscores the value of our NMS prediction model as a noninvasive tool to manage such variability. Future studies elucidating the relationship between these pre‐IVM factors and NMS, as well as analyses of cumulus expansion, cytoplasmic events, and chromosomal abnormalities, will be critical for optimizing individual culture systems.

In conclusion, extending IVM to 28 h significantly improved the fertilization competence of slow‐predicted NMS oocytes, thereby supporting our hypothesis that optimal IVF timing relative to nuclear maturation completion is crucial for successful embryo development. This improvement was evidenced by shortened first cleavage timing, suggesting potential enhancement of implantation competence. The extended IVM duration resulted in decreased expression of key developmental genes (*NANOG* and *OCT4*) in blastocysts, which likely reflects accelerated developmental progression rather than compromised embryo quality. Interestingly, *SOX2* expression decreased only in embryos from fast‐predicted NMS oocytes following extended IVM, while remaining unchanged in embryos from slow‐predicted NMS oocytes. These differential molecular responses highlight that fast‐ and slow‐predicted NMS oocytes respond differently at the molecular level to extended IVM duration. Future studies directly assessing implantation competence through embryo transfer would be valuable to confirm the developmental benefits of individualized IVM protocols.

## Author Contributions

**Thomas Chia‐Tang Ho:** conceptualization, investigation, writing – original draft. **Takashi Tanida:** methodology, resources, writing – reviewing and editing. **Takashi Fujii:** methodology, writing – reviewing and editing. **Keisuke Koyama:** supervision, conceptualization, investigation, funding acquisition, writing – reviewing and editing.

## Conflicts of Interest

The authors declare no conflicts of interest.
